# Characterisation of urinary monocyte chemoattractant protein 1: Potential biomarker for patients with overactive bladder

**DOI:** 10.1080/2090598X.2019.1589932

**Published:** 2019-04-04

**Authors:** Bilal Farhan, Huiyi Chang, Ahmed Ahmed, Frank Zaldivair, Gamal Ghoniem

**Affiliations:** a Department of Urology, University of California, Irvine, CA, USA; b Department of Urology, Aswan University, Aswan, Egypt

**Keywords:** Monocyte chemoattractant protein 1, overactive bladder, urinary cytokines

## Abstract

**Objective**: To investigate urinary monocyte chemoattractant protein 1 (MCP-1) as a potential marker for idiopathic overactive bladder (OAB). This is a quantitative measurement of urinary MCP-1 to establish baseline normal values that could help in future index studies. Normalised urinary MCP-1 levels are measured in female patients with OAB and aged-matched controls. Severity of OAB symptoms is correlated to normalised urinary MCP-1 levels.

**Patients and methods**: Urinary MCP-1 levels were measured in 29 female patients with OAB and 10 normal female controls. The patients with OAB were either newly diagnosed or off any OAB oral therapy for at least 2 weeks. OAB symptoms were assessed using validated OAB questionnaires. Urinary MCP-1 levels were measured using enzyme-linked immunosorbent assay and normalised by urinary creatinine (Cr) levels.

**Results**: The baseline urinary MCP-1 levels in female patients with OAB were significantly higher than those of the controls, at a mean of 210.25 vs 48.02 pg/mg Cr (*P* < 0.001). Patients who had severe OAB bother symptoms had higher levels of urinary MCP-1 (*r* = 0.03), also patients with OAB-wet had higher levels of urinary MCP-1, at a mean (SEM) of 209.25 (30.5) vs OAB-dry 185.25 (10) pg/mg Cr (*P* < 0.001).

**Conclusion**: Urinary MCP-1 levels were higher in female patients with idiopathic OAB. The close association of urinary MCP-1 and OAB bother severity symptoms and OAB-wet suggest that inflammation plays a major role in the pathophysiological mechanisms underlying the sensitisation of bladder afferent nerves. Establishing urinary MCP-1 levels in patients with OAB hopefully will help future studies to confirm the correlation as a baseline and changes with treatments.

**Abbreviations**: BMI: body mass index; Cr: creatinine; MCP-1: monocyte chemoattractant protein 1; OAB: overactive bladder; OAB-q: Overactive Bladder Questionnaire; PPBC: Patient Perception of Bladder Condition; UI: urinary incontinence

## Introduction

Overactive bladder (OAB) is a group of symptoms condition of urinary urgency, usually with frequency and nocturia, with or without urgency urinary incontinence (UI) [1]. Although the aetiology of OAB is currently still being researched, several recent studies have proposed that bladder urothelial dysfunction, with overexpression of sensory receptors, abnormal function of suburothelial interstitial cells, as well as increased excitability of detrusor muscles could play a role in the underlying pathophysiology of OAB [2]. Chronic inflammation has been found in bladder biopsies from patients with neurogenic bladder and idiopathic OAB [3]. Most recent studies of urinary chemokines in patients with OAB have shown elevated monocyte chemotactic protein 1 (MCP-1) and inflammatory proteins compared with controls, suggesting that chronic inflammation is a key feature in OAB [4–6]. Many studies have shown that urinary MCP-1 is expressed in a variety of different inflammation and chronic pain conditions [6].

Chemokines are classified into four subfamilies, i.e. CXC, CC, CX3C and XC. MCP-1/CCL2 is a member of the C-C chemokine family, and a potent chemotactic factor for monocytes. MCP-1 is produced by a variety of cell types, either constitutively or after induction by oxidative stress, cytokines, or growth factors [7].

In the present pilot study, we investigated urinary MCP-1 levels in women with OAB symptoms with respect to OAB subtypes and severity of symptoms.

## Patients and methods

After Institutional Review Board approval, adult women, diagnosed with OAB, and age-matched healthy volunteers without LUTS were recruited between October 2014 and March 2018. All participants signed an informed consent. All patients with OAB symptoms underwent history, physical examination, and urine analysis. Additional tests were done when appropriate, such as urine culture, uroflowmetry, and bladder scan for residual urine. The newly diagnosed patients with OAB were verified by 3-day voiding diary and only patients with a daily frequency of >8 episodes of urgency/day (OAB-dry) or urgency UI ≥1 episode/day (OAB-wet) were diagnosed as having OAB. Patients that had already been diagnosed with OAB had to have stopped taking OAB medications for at least 2 weeks prior to enrolment.

Patients with neurogenic bladder, BOO, urinary stones, tumours or UTI at the recruitment were excluded. Additionally, females with urogenital atrophy, severe stress UI, vaginitis or active genital disease, positive bacterial culture or a post-void residual urine volume of >150 mL were excluded. Patients were also excluded if they had any other condition that, in the opinion of the investigator, made them unsuitable for inclusion. Age-matched healthy volunteers (controls) were recruited from hospital staff and had no significant LUTS or pain (AUA Symptom Index <7), no previous diagnosis of OAB, and no evidence of infection based on a negative nitrite dipstick test and symptoms. Mid-stream urine samples were collected at the time of enrolment as a baseline and centrifuged at 3000 ***g*** for 10 min at 4°C to collect the supernatant, and 1.5 mL aliquots were preserved at – 80°C. Concurrently, 3 mL urine was taken to measure the urinary creatinine (Cr), in order to normalise the urinary MCP-1 with the urinary Cr level. No specific dietary restrictions during urine collection were applied.

Urinary MCP-1 levels were measured using R & D Systems Biotech ELISA Kit (Cat# DCP00; R & D Systems, Minneapolis, MN, USA). This kit is validated to perform well in multiple human matrices. The CCL/MCP-1 kit is intended to determine the quantitative levels of CCL2/MCP-1 in human urine by an enzyme immunoassay. This test is a 3.5–4.5 h solid-phase ELISA designed to measure MCP-1 in cell culture, serum, plasma, and urine. All reagents were prepared as specified by the manufacturer’s instructions, which includes well washing of the samples followed by incubation for 30 min at room temperature by addition of 200 μL substrate into each well. Finally, a stop solution was added to the wells and then optical density of each well was assessed using a microplate reader set at 450 nm. All measurements were performed in duplicate and the average was used to subtract from the zero standard optical density. The total urinary MCP-1 levels were further normalised by the concentration of urinary Cr. The levels of urinary MCP-1 were expressed as picogrammes (pg) per milligramme (mg) of Cr for both the controls and patients with OAB.

All data are presented as means (standard error of the mean, SEM). Statistical differences in mean levels of normalised urinary MCP-1 in patients with OAB, and controls, OAB-wet, OAB-dry, as well as age and body mass index (BMI) were compared using one-way ANOVA and paired *t*-tests between subgroups of two means at their baselines. Pearson’s correlation was used for analysing the association between normalised urinary MCP-1 levels and the OAB symptoms bother severity using the Overactive Bladder Questionnaire (OAB-q) and Patient Perception of Bladder Condition (PPBC) questionnaire. All statistical analyses were performed using the IBM Statistical Package for the Social Sciences (SPSS®) version 20.0 (SPSS Inc., IBM Corp., Armonk, NY, USA). A *P* < 0.05 was considered statistically significant.

## Results

In all, 29 women with OAB (18 women with OAB-wet, 11 with OAB-dry) and 10 controls were enrolled. The mean (SEM) age was 60.45 (9) years for the OAB group and 55.2 (12) years for the control group (*P *= 0.153). There was also no significant difference in the mean age between women with OAB-dry and OAB-wet (*P *= 0.223), as well as compared to the controls (*P *= 0.236). The baseline urinary MCP-1 levels for female patients with OAB were significantly higher than those in controls, at a mean of 210.25 vs 48.02 pg/mg Cr (*P* < 0.001). Additionally, the normalised urinary MCP-1 levels were significantly highest in patients with OAB-wet (Table 1). There was no significant difference in the mean BMI between patients with OAB-dry and OAB-wet (*P = *0.214).

**Table 1.** Patients with OAB and control subjects, OAB-wet, OAB-dry, age, and BMI.

		OAB (*n* = 29)		
Variable, mean (SEM)	Control (*n* = 10)	OAB-wet (*n* = 18)	OAB-dry (*n* = 11)	*P* (ANOVA)
Urinary MCP-1, pg/mg Cr	48.02 (9)	209.25 (30.5)	185.25 (10)	0.001
Age, years	55.2 (12)	58.4 (8)	62.5 (10)	0.236
BMI, kg/m^2^	28.3 (0.2)	30.6 (2.7)	29.4 (2)	0.626

**Figure 1.**
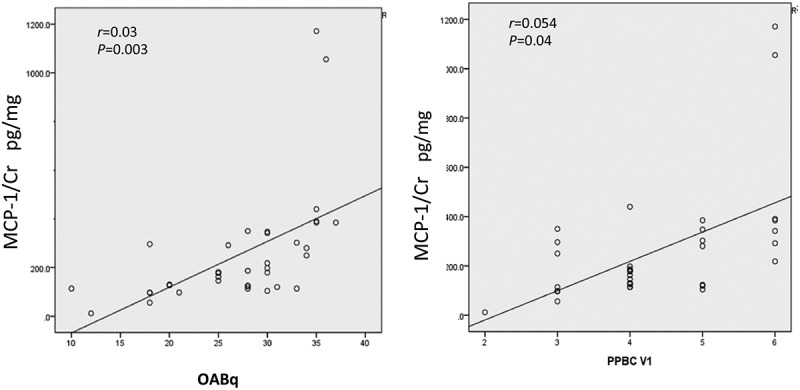
Relationship between mean urinary MCP-1/Cr levels and OAB symptoms using Spearman correlation coefficient.

Using Pearson’s correlation, normalised urinary MCP-1 levels significantly correlated with the severity OAB symptoms on the OAB-q (Figure 1; *r* = 0.03, *P *= 0.003).

## Discussion

Much evidence suggests that inflammatory processes are involved in OAB pathogenesis. Ghoniem et al. [8] proposed the hypothesis of local inflammation as a cause and to play a central role in the aetiology of OAB. Previous studies have shown that increased urinary levels of MCP-1 are evidence of bladder inflammation [9]. In the present pilot study, we investigated urinary MCP-1/Cr levels in patients with OAB using a quantitative method (Quantikine® Human MCP-1 Immunoassay, ELISA designed) and compared it to healthy age-matched volunteers (controls). The present results provide evidence that women with OAB have higher levels of urinary MCP-1, especially in those with OAB-wet, suggesting that chronic inflammation results in greater expression of urinary MCP-1; whilst in contrast OAB-dry could be a mixture of urothelial dysfunction and increased bladder sensation such as detrusor overactivity. This appears to indicate that MCP-1 could be a possible marker to differentiate between OAB subtypes. The other reason could be the result of higher degree of inflammation and higher percentage of detrusor overactivity.

There were close correlations between the urinary MCP-1/Cr levels and the severity of OAB symptoms. This finding is the main strength of our present study, suggesting the ability to predict the severity of symptoms with levels of urinary MCP-1/Cr. The mechanism through which urinary levels of MCP-1 increase in patients with OAB is not yet well establish. Stretching of the bladder smooth muscle cells increases the expression of mRNA leading to increased expression and secretion of urinary cytokines [10]. Further investigations and studies are needed to confirm the present results, as well as the precise mechanisms involved.

A limitation of the present pilot study was the small cohort of controls and patients with OAB. A larger sample size will enable a more detailed study with follow-up of the response of urinary MCP-1 to OAB treatments.

Overall, our present findings may serve as a basis to study the pathogenesis of OAB in the context of chronic systemic inflammation. The level of urinary MCP-1 might serve as a potential tool for studying the progression, severity, and to assess the therapeutic effects of treatment, allowing a better understanding of the pathophysiology of the OAB syndrome.

In conclusion, urinary MCP-1 was significantly higher in women with OAB-dry and OAB-wet. Higher urinary MCP-1 in OAB-wet could be related to a local inflammatory disorder, which is unrelated to obesity and ageing. This finding may help to understand the pathogenesis of OAB in the context of chronic inflammation, which might give the light at the end of the tunnel for treatment of patients with OAB syndrome.
